# Reproducibility and inter-observer variability of velocity and 3D wall shear stress derived from 4D flow MRI in the healthy aorta

**DOI:** 10.1186/1532-429X-17-S1-P74

**Published:** 2015-02-03

**Authors:** Pim van Ooij, Alexander L Powell, Wouter V Potters, Alex J Barker, Michael Markl

**Affiliations:** 1 Radiology, Northwestern University, Chicago, IL, USA; 2 Radiology, Academic Medical Center, Amsterdam, Netherlands; 3 Biomedical Engineering, Northwestern University, Chicago, IL, USA

## Background

4D flow MRI is a powerful tool to measure time-resolved three-directional blood flow in the pathological and healthy aorta. The 3D aortic velocity measured with 4D flow MRI allows for derivation of 3D wall shear stress (WSS), which is believed to be related to vessel wall remodeling and possibly an important biomarker for aortopathy. However, it is still unclear how variability of the 3D vessel segmentation needed for 3D WSS calculation and/or scan vs. rescan changes in the underlying 4D flow MRI data can affect the reproducibility of the results. The aim of this study was therefore to investigate the test-retest reproducibility and inter-observer variability of 4D flow derived 3D velocity fields and 3D WSS in the thoracic aorta.

## Methods

Fourteen healthy controls underwent two (test and retest) 4D flow MRI examinations separated by 16±3 days. Peak systolic velocity and WSS were calculated for both scans and compared individually. Cohort-averaged systolic 3D velocity fields and 3D WSS maps for test and retest cohorts were generated by: 1) creation of a shared aorta geometry by rigid co-registration of all aortas scanned in the test or retest session and determination of the maximum overlap of all aortas. 2) Interpolation of all individual systolic 3D velocity fields and 3D WSS vectors to the shared geometry and subsequent averaging of velocity and WSS over the cohort. The individual subjects and the cohort-averaged maps were compared by voxel-by-voxel Bland-Altman and orthogonal regression analysis. The mean difference and limits of agreement (LOA) were expressed as a percentage of the mean velocity and WSS in the entire aorta. Inter-observer variability was calculated for WSS.

## Results

Individual Bland-Altman analysis revealed moderate differences (~10%) and LOA (~60%) between test and retest for both velocity and WSS. Orthogonal regression analysis showed moderate agreement between test and retest (Slope: ~1.20±0.21 for velocity and WSS, Pearsons ρ: 0.74±0.06 for velocity and 0.64±0.12 for WSS). The test and retest-averaged velocity and WSS maps showed small differences (~6%) and LOA (~22%) and good agreement (Slope: 1.10, ρ: 0.91). For WSS inter-observer variability the difference was 3%, LOA: 38%, Slope: 1.06±0.20 and ρ: 0.85±0.11.

## Conclusions

Peak systolic velocity and WSS derived from 4D flow MRI are reproducible quantities with low inter-observer variability in this cohort of healthy volunteers.

## Funding

AHA grant 14POST20460151;

NIH grant K25HL119608;

NIH NHLBI grant R01HL115828;

Dutch Technology Foundation (STW) Carisma Grant 11629.

**Figure 1:**
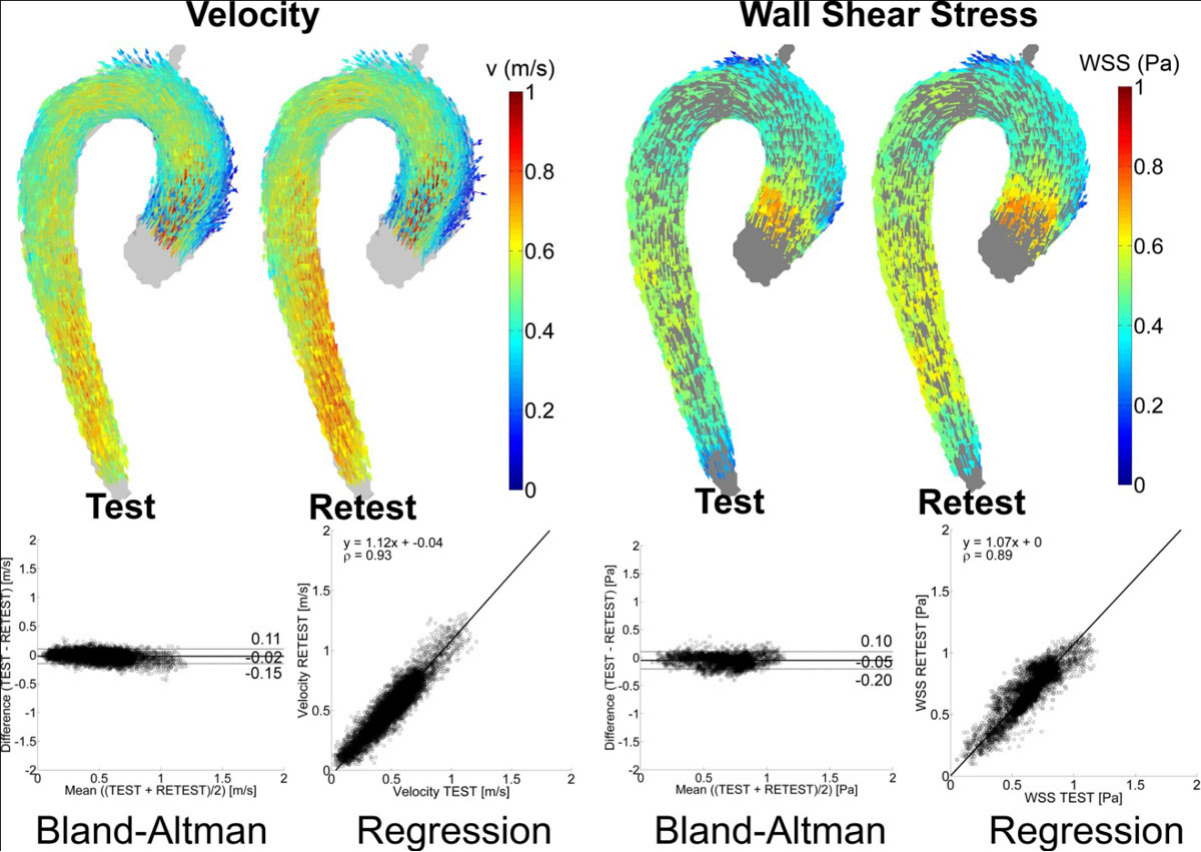
**Three-dimensional test- and retest-averaged peak systolic velocity and WSS maps (top row).** Results of the Bland-Altman and orthogonal regression analyses (bottom row) for the maps shown in the top row.

